# Personal

**Published:** 1917-05

**Authors:** 


### PERSONAL

Dr. Edgar R. McGuire of Buffalo has been appointed Prof. of Surgery in the University of Buffalo.

Dr. George Edward Fell of Buffalo is at 846 Webster Avenue, Chicago.

Dr. Ernest Milton Watson announces the opening of offices in the Electric Building, Buffalo, practice limited to Urology.

Drs. Wm. M. and Edward H. Mehl of Buffalo announce the removal of their office to 417 Franklin Street. Practice limited to Diseases of the Eye, Ear, Nose and Throat.

Dr. John L. Heffron of Syracuse, who has been spending several weeks in Florida to recuperate his health, has fully recovered and has returned to his home.

Dr. Karl F. Eschelman of Buffalo announces the removal of his office to 758 Elmwood Avenue on April 15.

Dr. A. L. Benedict of Buffalo would greatly appreciate information as to sites of Indian villages, burying grounds and similar recent discoveries.

Major W. W. Quinton, M. D., who has been for several years a resident of Buffalo, will leave shortly for Cambridge, Md.

